# Technical note on the exploration of COVID-19 in autopsy material

**DOI:** 10.1136/jcp-2022-208525

**Published:** 2023-01-30

**Authors:** Matthew Phillip Humphries, Victoria Bingham, Fatima Abdullah Sidi, Stephanie Craig, Beatrize Lara, Hesham El-daly, Nicole O'Doherty, Perry Maxwell, Claire Lewis, Stephen McQuaid, James Lyness, Jacqueline James, David R J Snead, Manuel Salto-Tellez

**Affiliations:** 1 Precision Medicine Center of Excellence, Queen's University Belfast, Belfast, UK; 2 National Pathology Imaging Cooperative, Leeds Teaching Hospitals NHS Trust, Leeds, UK; 3 University Hospitals Coventry and Warwickshire NHS Trust, Coventry, UK; 4 Northern Ireland State Pathologist's Department, Belfast, UK; 5 Northern Ireland Molecular Pathology Laboratory, Queen's University Belfast, Belfast, UK; 6 The Patrick G Johnston Centre for Cancer Research, Queen’s University, Northern Ireland Biobank, Belfast, UK; 7 Pathology, University Hospitals Coventry and Warwickshire NHS Trust, Coventry, UK; 8 Division of Molecular Pathology, The Institute of Cancer Research, London, UK

**Keywords:** Autopsy, COVID-19, Pathology, Molecular, MOLECULAR BIOLOGY

## Abstract

Interrogation of immune response in autopsy material from patients with SARS-CoV-2 is potentially significant. We aim to describe a validated protocol for the exploration of the molecular physiopathology of SARS-CoV-2 pulmonary disease using multiplex immunofluorescence (mIF).

The application of validated assays for the detection of SARS-CoV-2 in tissues, originally developed in our laboratory in the context of oncology, was used to map the topography and complexity of the adaptive immune response at protein and mRNA levels.

SARS-CoV-2 is detectable in situ by protein or mRNA, with a sensitivity that could be in part related to disease stage. In formalin-fixed, paraffin-embedded pneumonia material, multiplex immunofluorescent panels are robust, reliable and quantifiable and can detect topographic variations in inflammation related to pathological processes.

Clinical autopsies have relevance in understanding diseases of unknown/complex pathophysiology. In particular, autopsy materials are suitable for the detection of SARS-CoV-2 and for the topographic description of the complex tissue-based immune response using mIF.

## Introduction

SARS-CoV-2, first reported in Wuhan (China) in December 2019, was declared a global pandemic by the WHO in March 2020.^1^

Following authoritative calls defending the potential key role of autopsy-based analyses in COVID-19,^2^ we present herein a model for a molecular physiopathological analysis of COVID-19 autopsy samples. We include validations of the following approaches in challenging formalin-fixed, paraffin-embedded (FFPE) autopsy materials: immunohistochemistry (IHC) and RNA in situ hybridisation detection of SARS-CoV-2 and multiplex analysis of the immune response in the COVID-19 pneumonic process, with digital pathology quantitation.

## Methods

### Materials

To validate COVID-19 detection, we used COVID-19-positive control FFPE macaque tissue from collaborators at Erasmus University in Rotterdam.^3^ To confirm the specificity of our tests, we analysed 64 lung biopsy and respiratory tract cytology samples, excluding COVID-19 pneumonia.^4^

**Figure 1:**
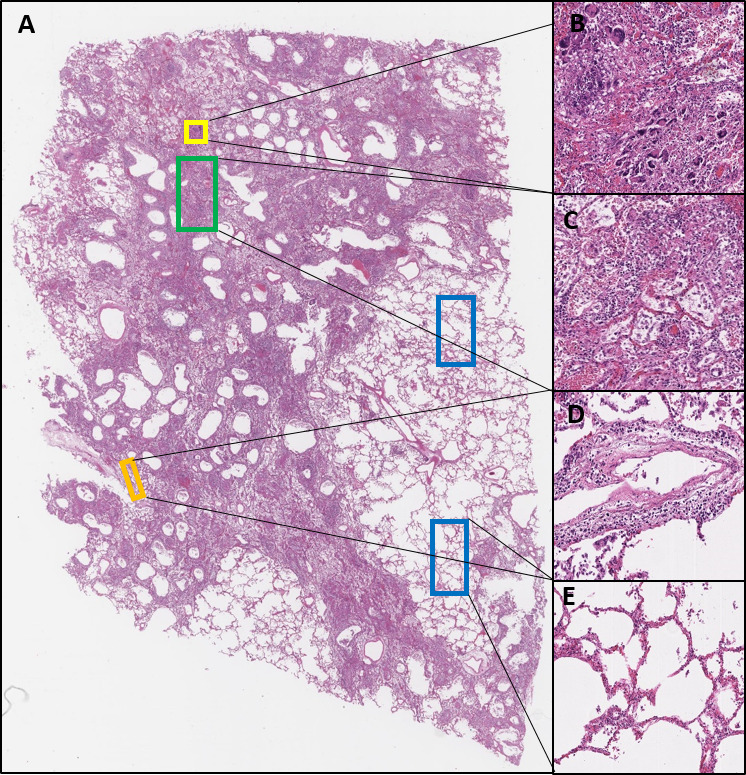
(A) Whole face H&E from COVID-19-positive autopsy case 2. (B) Illustration of an area of viral syncytial formation (yellow annotation), ×20; (C) long-standing diffuse alveolar damage with re-epithelisation (green annotation), ×20; (D) vascular inflammatory changes (orange annotation), ×20; and (E) minimal chronic interstitial inflammation (blue annotations), ×20.

In addition, we tested FFPE lung tissue taken at autopsy from two patients dying from pneumonia with positive COVID-19 PCR tests and from two patients who died of non-COVID-19-related pneumonia, with a time of disease development and autopsy performance well before December 2019. We refer to cases 1 and 2 as the two COVID-19 proven autopsy cases, while cases 3 and 4 represent patients who died with pneumonia (more than 6 months before the outbreak). The aetiology and lung pathology of each case is described in online supplemental data 1. A COVID-19-positive H&E is shown in figure 1, where the physiopathology of several areas are illustrated: overview (figure 1A), a viral syncitial formation (figure 1B), DAD with re-epithelisation (figure 1C), vascular inflammatory changes (figure 1D) and minimal chronic interstitial inflammation (figure 1E).

10.1136/jcp-2022-208525.supp1 Supplementary data

### In situ detection of SARS-CoV-2

Detection was conducted in the macaque animal model, the retrospective clinical samples pre-COVID-19 and in the four autopsy samples described. Three-micrometre-thick sequential sections were obtained from all blocks and stained for SARS-CoV-2 nucleoprotein IHC or V-nCoV2019-S RNAScope. Automated IHC was performed using a Leica BOND RX with a rabbit monoclonal antibody against SARS-CoV-2 nucleoprotein (40143-T62; Sino Biologicals, Pennsylvania, USA). IHC was optimised on known COVID-19-positive tissue.^3^ IHC required pretreatment with epitope retrieval solution 1 for 10 min at 100°C and a 1:2000 antibody dilution. Detection chemistry employed was BOND Polymer Refine Detection Kit (DS9800; Leica Biosystems, USA) or BOND Polymer Refine Red Detection Kit (DS9390, Leica Biosystems). RNAScope to detect viral mRNA was performed using a Leica Bond RX system using RNAscope 2.5 LS Probe to V-nCoV2019-S, nt: 21 631–23 303 (848568; Advanced Cell Diagnostics, USA). For assessment of RNA integrity, we used RNAscope 2.5 LS Positive Control Probe- Hs-UBC (312028, Advanced Cell Diagnostics). Detection chemistry employed was RNAscope 2.5 LSx Reagent Kit-BROWN (322100, Advanced Cell Diagnostics) or RNAscope 2.5 LS Reagent Kit-RED (322150, Advanced Cell Diagnostics). All sections were digitised using a Leica Aperio AT2 Scanner at ×40 and analysed qualitatively and quantitatively in silico via Xplore (Philips) or the open source digital image analysis software programme QuPath V.0.2.0.^5^

### Multiplex IHC fluorescence

Three-micrometer-thick sections were obtained from autopsy blocks and stained using two previously validated multiplex panels.^6 7^ Panel 1 assessed CD3 (T cells), CD4 (T-helper cells), CD20 (B cells) and CK (epithelial cells); panel 2 assessed PD-L1 (immune checkpoint marker), CD8 (cytotoxic T cells), CD68 (macrophages) and CK. Staining detection was performed using an Opal 7-Colour Automation IHC Kit (Akoya Biosciences, Marlborough, Massachusetts, USA). Antibody specifics and optimised retrieval methods for multiplex panels are detailed in online supplemental table S1. Opal fluorophores were used according to the manufacturer’s instructions. All multiplex slides were digitised using a Vectra Polaris scanner (Akoya Biosciences) at ×20.

### Digital image analysis

3,3′-Diaminobenzidine (DAB) scan files captured on a Lecia Aperio AT2 (.svs) or whole-slide Opal MOTiF fluorescence images captured on a Akoya Vectra Polaris (.qptiff) were imported in to QuPath. Following precise annotation transfer from H&Es to the DAB and multiplex immunofluorescent images, a rigorous quality control process was undertaken by an experienced image analyst and histopathologist to ensure no specious factors impacted extracted data, confirmed by a second reviewer, as previously reported.^6–8^

For multiplex immunofluorescence (mIF), cell detection was conducted using DAPI within annotated regions of interest (ROIs). Single-channel biomarker detection was carried out using positive cell detection features based on consensus thresholds across each panel using nuclear/cell Opal mean. Density per square millimetre data and overall positive cell count, expressed as a percentage, was extracted into Microsoft Excel.

## Results

### In situ detection of SARS-CoV-2

**Figure 2:**
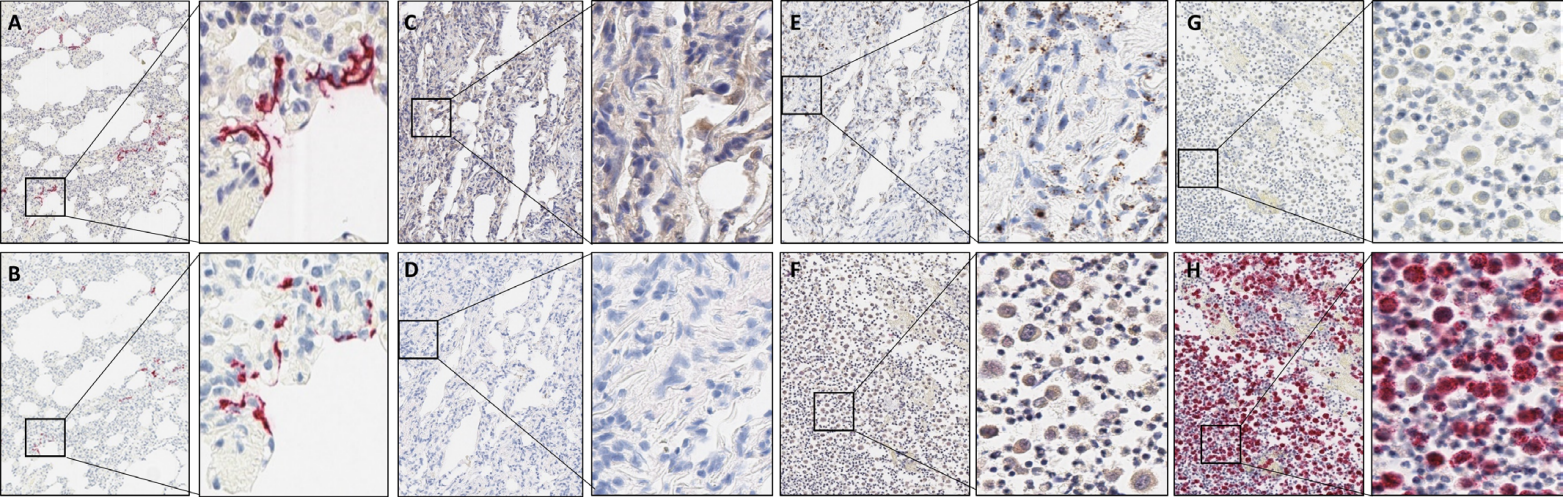
(A) Representative images showing SARS-CoV-2 nucleoprotein expression and (B) V-nCoV2019-S mRNA in FFPE macaque tissue. (C) Absence of SARS-CoV-2 nucleoprotein expression is depicted in lung biopsy FFPE and (F) lung cytology FFPE. (D) Absence of V-nCoV2019-S mRNA expression is depicted in lung biopsy FFPE and (G) lung cytology FFPE. (E) Positive control probe UBC mRNA expression is depicted in lung biopsy FFPE (reagent kit-BROWN) and (H) lung cytology FFPE (reagent kit-RED). All images are ×10 magnification, with exploded views at ×40. FFPE, formalin-fixed, paraffin-embedded.

Initially, we optimised COVID-19 IHC and RNAscope tests in positive control FFPE macaque tissue.^3^ Both tests were highly sensitive and specific in detecting SARS-CoV-2. SARS-CoV-2 was detected as expected by IHC and RNA-ISH in positive macaque control material (figure 2A,B). No evidence of SARS-CoV-2 by either IHC or RNA-ISH was seen in retrospective lung biopsy and lung cytology FFPE material (figure 2C,D,F,G). RNA integrity was confirmed with positive control probe UBC (figure 2E,H).

All 64 clinically non-COVID-19 lung biopsy or cytology samples were negative for SARS-CoV-2 by IHC or RNAScope. In 7/50 cytology cell blocks, there was no expression of the RNAScope positive control probe UBC.

**Figure 3:**
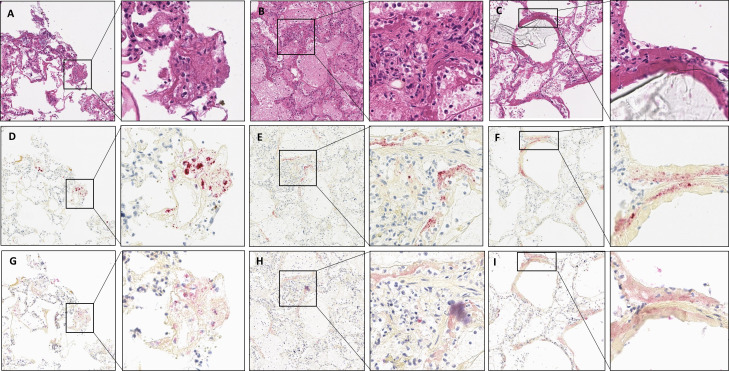
(A–C) Representative images showing H&E staining from COVID-19 autopsy case 1, (D–F) V-nCoV2019-S RNAScope and (G–I) SARS-CoV-2 nucleoprotein expression in three regions of FFPE autopsy tissue. All images are ×10 magnification, with exploded views at ×20. FFPE, formalin-fixed, paraffin-embedded.

COVID-19 RNA and protein signals were not present in non-COVID-19 autopsy cases (cases 3 and 4). As depicted in figure 3, in autopsy case 1, numerous, scattered viral signals were identified by both IHC and RNAScope probe. Autopsy case 2 showed no positive IHC or RNA-ISH signals.

To test our protocol for future analysis of inflammation associated with specific detectable portions of viral RNA or protein, we validated IHC and RNA-ISH detection in the context of DAB dual-plex and mIF, which was able to robustly detect SARS-CoV-2 in the macaque model and clinical autopsy 1 (online supplemental figure S1).

### Capturing the immune microenvironment with multiplex IHC fluorescence

**Figure 4:**
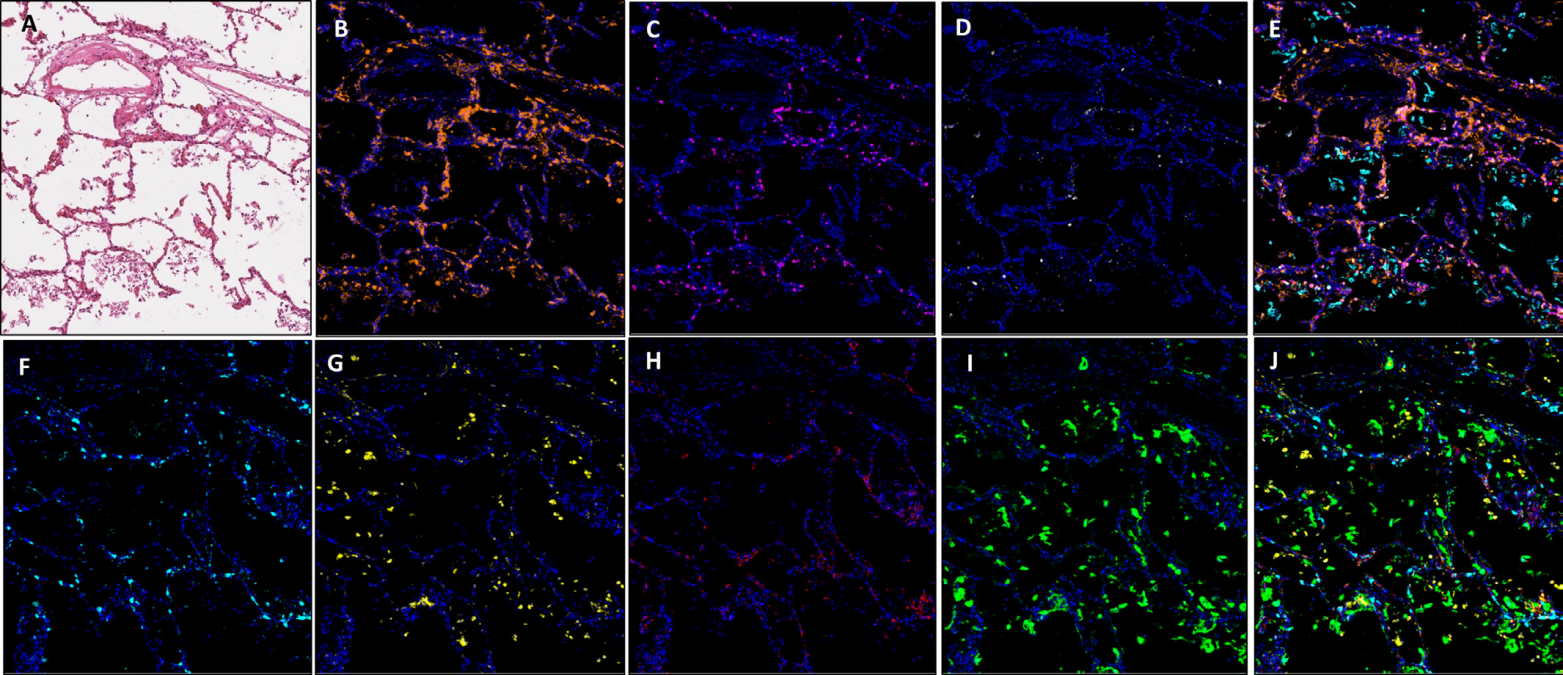
Images showing an area of minimal chronic interstitial inflammation in COVID-19 lung FFPE autopsy tissue case 1. (A) H&E; (B) CD3 (2GV6, orange); (C) CD4 (SP35, purple); (D) CD20 (L26, white); (E) composite CD3/CD4/CD20/CK and DAPI; (F). CD8 (C8/144B, aqua); (G). CD68 (514H12, yellow); (H) PD-L1 (SP263, red); (I) CK (AE1/AE3, green); and (J) composite CD8/CD68/PD-L1/CK and DAPI. All images are ×20 magnification. FFPE, formalin-fixed, paraffin-embedded.

**Figure 5:**
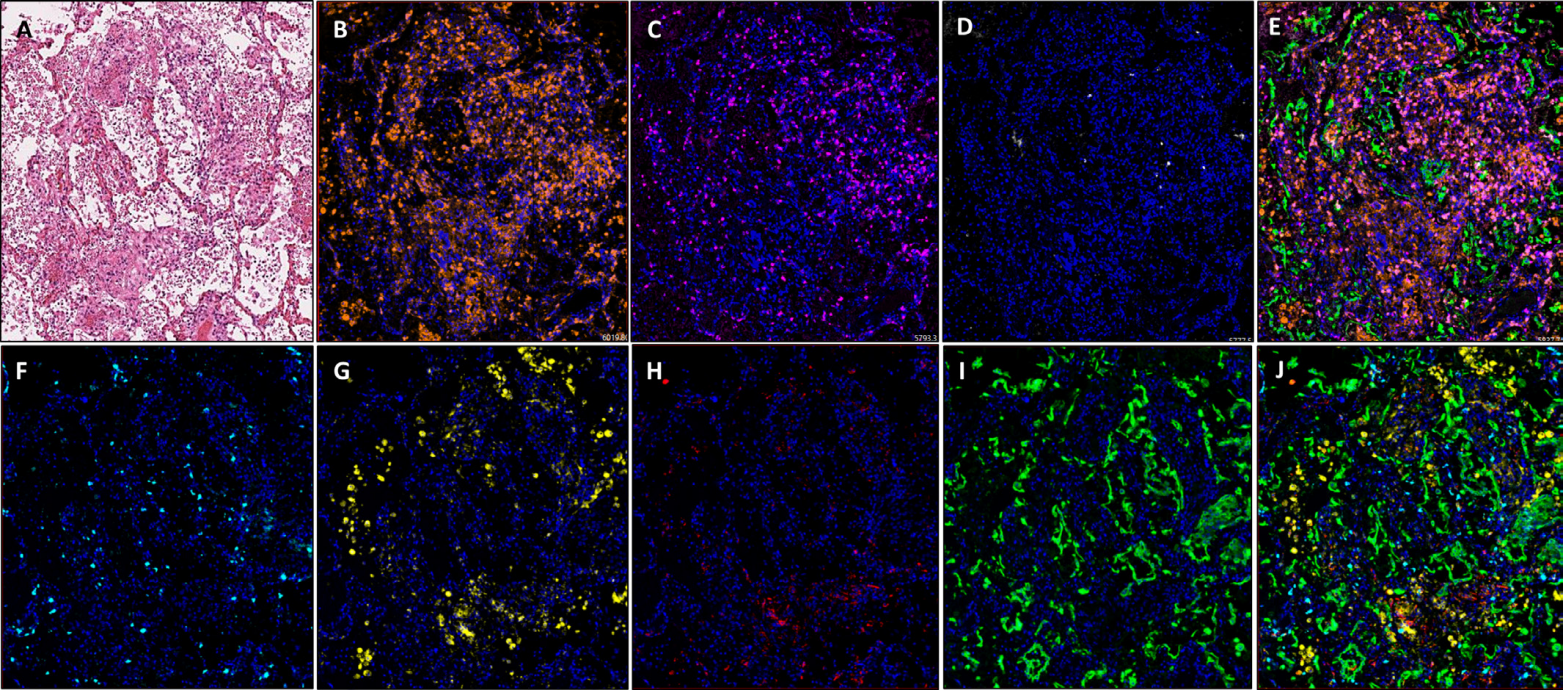
Images showing diffuse damage in COVID-19 lung FFPE autopsy tissue. (A) H&E, (B). CD3 (2GV6, orange); (C) CD4 (SP35, purple); (D) CD20 (L26, white); (E) composite CD3/CD4/CD20/CK and DAPI; (F) CD8 (C8/144B, aqua); (G) CD68 (514H12, yellow); (H) PD-L1 (SP263, red); and (I) CK (AE1/AE3, green); and (J) composite CD8/CD68/PD-L1/CK and DAPI. All images are ×20 magnification. FFPE, formalin-fixed, paraffin-embedded.

Application of multiplex immunofluorescent panels in autopsy material, previously optimised for our immune oncology studies,^6 7^ enabled us to delineate a range of immune cell phenotypes within COVID-19-positive material. To depict this visually, we chose two ends of the spectrum, namely, (1) an area of minimal chronic interstitial inflammation (figure 4) and (2) a heavily inflammed area (figure 5). Here we specifically quantifed total cell number and denisty per square millimetre for each biomarker and were able to analyse comparative expression of cell types using our robust digitial pathology workflow.

**Figure 6:**
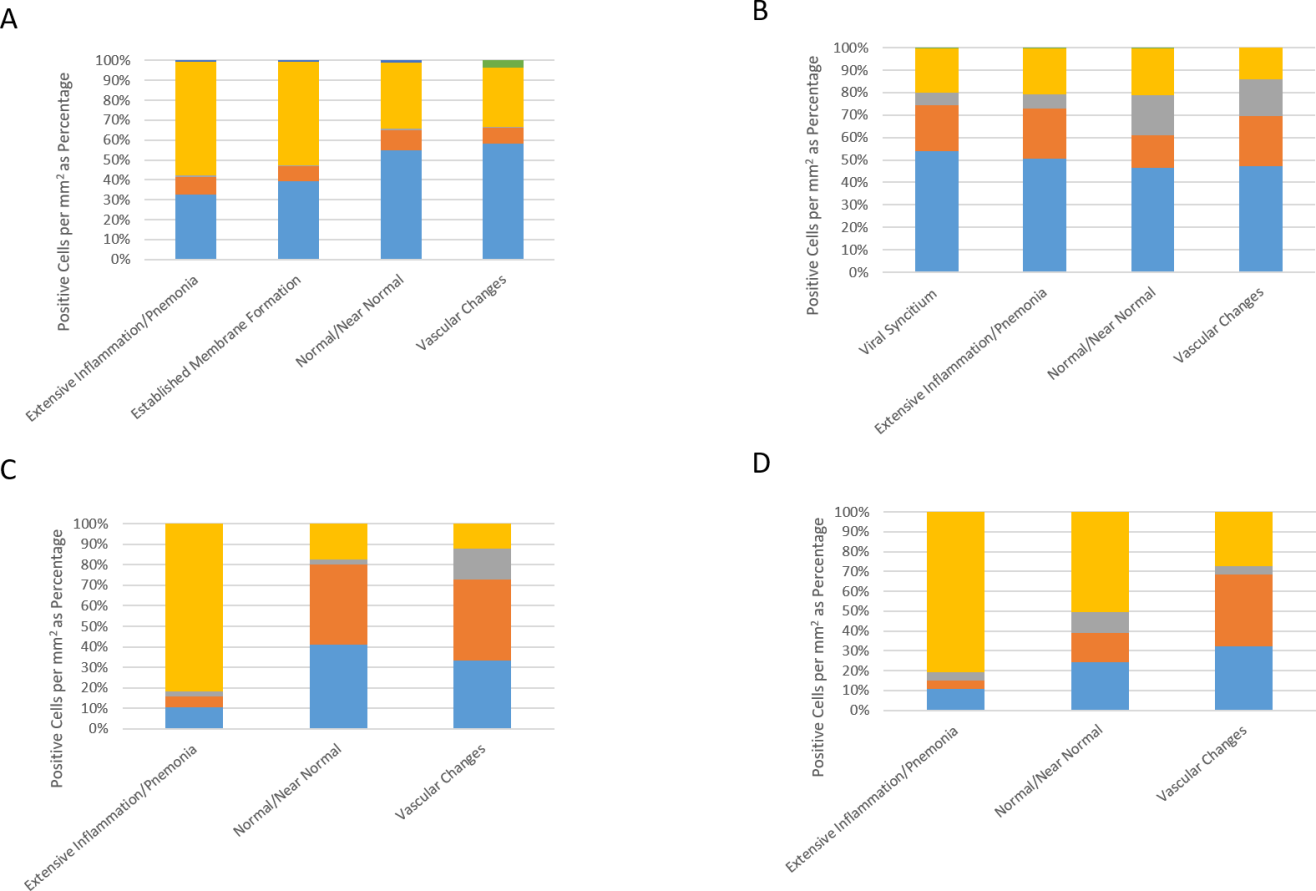
Bar graphs representing the density of immune cells per square millimetre represented as a percentage in COVID-19-positive and COVID-19-negative cases. Areas of normal/near normal, viral syncytium, extensive inflamation/pneumonia, vascular changes and established hyaline membrane formation are shown for (A) case 1, (B) case 2, (C) case 3 and (D) case 4. CD3, blue; CD4, orange; CD8, grey; CD68, yellow; CD20, green.

In comparison to normal/near normal areas within COVID-19-positive case 2, ROIs representing diffuse damage contained a markedly increased expression of CD3, CD4 and CD68, in terms of density per square millimetre (online supplemental file 1). It was observed that while there was an increase in overall cell density in the ROI representing diffuse damage, the percentage distribution between ROIs was similar (figure 6). The exception to this observation was a marked reduction in overall percentage of CD8-positive cells, which fell from 17.8% in the minimal chronic interstitial inflammation ROI to 5.7% in the areas with diffuse inflammatory damage (figure 6B).

As expected, areas of extensive pneumonia and oedema have a high density of CD68 expressing macrophages. In areas displaying vascular chages in all samples, the highest expressed markers in our panel were CD3 and CD4. In all ROIs, density of CD8-positive cells was higher in areas of normal/near normal when compared with areas of substantial change, that is, viral syncytium, extensive inflamation/pneumonia, vascular changes and established hyaline diffuse damage membrane formation (figure 6). These changes in immune cell phenotype across ROIs was as much as fivefold different (online supplemental figure S2).

The diversity of phenotypes when comparing SARS-CoV-2 positive cases with non- SARS-CoV-2 cases, in ROIs of vascular change is striking. Across all immune cells assessed (CD3, CD4, CD8, CD68 and D20), SARS-CoV-2-positive cases had a far higher total positive cell density compared with non-SARS-CoV-2 cases (positive cases 1 and 2: 909.6 mm^2^ and 801.23 mm^2^, respectively. Non-SARS-CoV-2 cases 3 and 4: 100.1 and 140.1 mm^2^, respectively).

Taken together, the T-cell and macrophage expressions seen within the vascular change ROIs in SARS-CoV-2 versus non- SARS-CoV-2 are also contrasting. In SARS-CoV-2-positive cases, there was a combined CD3 density of 904.62 mm^2^ (527.6 and 377.01 mm^2^ in each case), yet in both non-SARS-CoV-2 cases, the density of CD3 was greater than 10 times lower at 78.41 mm^2^ (33.36 and 45.05 mm^2^ in each case). This dichotomy in inflammation in SARS-CoV-2 versus non- SARS-CoV-2 cases was observed across immune markers: CD4 (255.48 mm^2^ vs 90.11 mm^2^), CD8 (113.52 mm^2^ vs 21.21 mm^2^) and CD68 (383.51 mm^2^ vs 50.45 mm^2^).

We only observed an identifiable increase in expression of B cells in one SARS-CoV-2-positive case. Case 1, in comparison to areas of normal/near normal (normal: 5.9 mm^2^, vascular change 33.7 mm^2^) showed a 570% increase in expression. While this was not observed in case 2, this may be explained by a contrasting clinical time course of COVID-19 between each patient. CD20 expression was not seen in non-COVID-19 cases. For each biomarker in the two multiplex panels, individual cells were identified with the same sensitivity and specificity as we have observed in lung cancer tissue studies.

## Discussion

The diagnostic testing of COVID-19 is primarily PCR based on upper respiratory tract samples, or ELISA-type blood based.^9^ Our validation suggests tissue hybridisation detection of SARS-CoV-2 could be reliably used but may be sensitive to disease stage. This may explain the lack of detectable in situ SARS-CoV-2 hybridisation signal in one of the two COVID-proven autopsy samples tested.

The validation of multiplexing analyses, originally developed in our laboratory in the context of oncology,^7 8 10^ enables the opportunity to illuminate the immune microenvironment of COVID-19 related changes. Our results indicate that there is significant variation in the immune reaction in different areas within affected lungs. In addition, our digital pathology approach allows identification of these changes of up to fivefold. While our observations indicate increased immune cells in COVID-19-positive autopsy cases in comparison to samples from non-COVID-19 autopsies, we cannot conclude that viral infection by COVID-19 ultimately leads to an increase in immune response over and above other viral infections. Indeed, studies have demonstrated that T-cell suppression may be due to increased immune checkpoints (CTLA4, PD-L1 and IDO-1),^9^ as well as those detected in active disease in soluble blood (TIM3 and LAG3). The being said, immune response to COVID-19 infection remains poorly understood, and our data, while limited, is in line with others, which is suggestive of a heightened immune response,^11^ while several conflicting studies demonstrate T-cell exhaustion.^12^ Very few published studies have used mIF to help describe the pathology of COVID-19, primarily to describe the immune response associated with vasculature.^13^

This works demonstrates a proof of principle methodology to capture the immune microenvironment in COVID-19-positive tissues. Further, we establish that the immune microenvironment in COVID-19 autopsy tissue is detectable and quantifiable. Our data indicate that quantification of the immune response to COVID-19 can be achieved within a routine molecular pathology laboratory and, once adapted to clinical samples other than resections (such as materials form biopsies and cytologies), would lend itself to screening of routine diagnostic biopsy samples.

## References

[1] World Health Organization . Coronavirus disease 2019 (COVID-19) situation report 67. Available: https://www.who.int/emergencies/diseases/novel-coronavirus-2019/situation-reports [Accessed 30 Mar 2020].

[2] Barth RF , Xu X , Buja LM . A call to action: the need for autopsies to determine the full extent of organ involvement associated with COVID-19. (1931-3543 (electronic)). 10.1016/j.chest.2020.03.060 PMC7151356 32283063

[3] BA-OX R , Kuiken TA-O , Herfst SA-O . Comparative pathogenesis of COVID-19, MERS, and SARS in a nonhuman primate model. (1095-9203 (electronic)). 10.1126/science.abb7314 PMC7164679 32303590

[4] Lewis C , McQuaid S , Clark P , et al . The Northern Ireland Biobank: a cancer focused Repository of science. Open J Bioresour 2018;5. 10.5334/ojb.47

[5] Bankhead P , Loughrey MB , Fernández JA , et al . QuPath: open source software for digital pathology image analysis. Sci Rep 2017;7:16878. 10.1038/s41598-017-17204-5 29203879 PMC5715110

[6] Viratham Pulsawatdi A , Craig SG , Bingham V , et al . A robust multiplex immunofluorescence and digital pathology workflow for the characterisation of the tumour immune microenvironment. Mol Oncol 2020;14:2384–402. 10.1002/1878-0261.12764 32671911 PMC7530793

[7] Humphries MP , Bingham V , Abdullahi Sidi F , et al . Improving the diagnostic accuracy of the PD-L1 test with image analysis and multiplex hybridization. Cancers 2020;12. doi:10.3390/cancers12051114. [Epub ahead of print: 29 Apr 2020]. PMC7281311 32365629

[8] Humphries MP , Craig SG , Kacprzyk R , et al . The adaptive immune and immune checkpoint landscape of neoadjuvant treated esophageal adenocarcinoma using digital pathology quantitation. BMC Cancer 2020;20:500. 10.1186/s12885-020-06987-y 32487090 PMC7268770

[9] Dinnes J , Deeks JJ , Adriano A , et al . Rapid, point-of-care antigen and molecular-based tests for diagnosis of SARS-CoV-2 infection. Cochrane Database Syst Rev 2020;8:CD013705. 10.1002/14651858.CD013705 32845525 PMC8078202

[10] Abdullahi Sidi F , Bingham V , Craig SG , et al . PD-L1 multiplex and quantitative image analysis for molecular diagnostics. Cancers;13:29. 10.3390/cancers13010029 PMC7796246 33374775

[11] Zhang B , Zhou X , Qiu Y . Clinical characteristics of 82 death cases with COVID-19. medRxiv 2020:2020.2002.2026.20028191.

[12] Diao B , Wang C , Tan Y , et al . Reduction and functional exhaustion of T cells in patients with coronavirus disease 2019 (COVID-19). Front Immunol 2020;11. 10.3389/fimmu.2020.00827 PMC7205903 32425950

[13] Dorward DA-O , Russell CA-O , IH U . Tissue-Specific immunopathology in fatal COVID-19. (1535-4970 (electronic)). 10.1164/rccm.202008-3265OC PMC7874430 33217246

