# Supplementary material for: Technical note on the exploration of COVID-19 in autopsy material
Source: J Clin Pathol. 2023 Jan 30;76(6):418–23. doi: 10.1136/jcp-2022-208525 (PMC10313984; doi:10.1136/jcp-2022-208525)
Supplement: Supplementary data [file jcp-2022-208525supp001.pdf]

Supplementary Data 1

Case 1 had a short clinical course of 5 days. This late 40s lady had a history of asthma and feeling generally unwell for several days. She was a heath care worker who felt the symptoms of COVID-19 (pyrexia and cough) and was self isolating. She was found collapsed on her bedroom floor and, despite resuscitation attempts, was pronounced dead on the same day. The autopsy identified pleural and pericardial effusion, as well as a lung histology dominated by extensive hyaline membrane disease /diffuse alveolar damage (DAD) and pulmonary oedema. Virology tests proved positive for SARS-CoV-2 on both the swabs from the nose and mouth and the lower bronchial tree.

Case 2 is an late 80s gentleman admitted to hospital with cough and breathlessness. Subsequently diagnosed with COVID-19 by PCR. During the admission he developed acute kidney failure and a deep vein thrombosis. Managed on the ward with oxygen and fluid balance and doxycycline. Entered into recovery trial, but his condition deteriorated suddenly and died 13 days post admission. The autopsy found pneumonia, left ventricular hypertrophy, coronary artery atherosclerosis and a swollen left leg in keeping with deep vein thrombosis. The lung histology showed DAD of fibrotic stage, focal bronchopneumonia, isolated foci of thrombosis in small capillary sized vessels and focal re-epithelialization of alveolar spaces with squamous metaplasia. Figure 1 illustrates the complex pathology of this case, with an overall impression of the lung damage (1A); evidence of florid viral syncytial formation (1B); end-stage diffuse alveolar damage with re-epithelization (1C); vascular changes (1D); and a relatively unaffected area of lung parenchyma for comparison (1E).

Case 3 was a diabetic patient with mental health issues, found dead at home and with macroscopic and histological evidence of pneumonia on autopsy.

Case 4 presented to the forensic pathology team with past medical history of pneumonia, anxiety and depression. At autopsy he had an acute pneumonia and emphysema, confirmed by histology, in an overall state of malnourish.

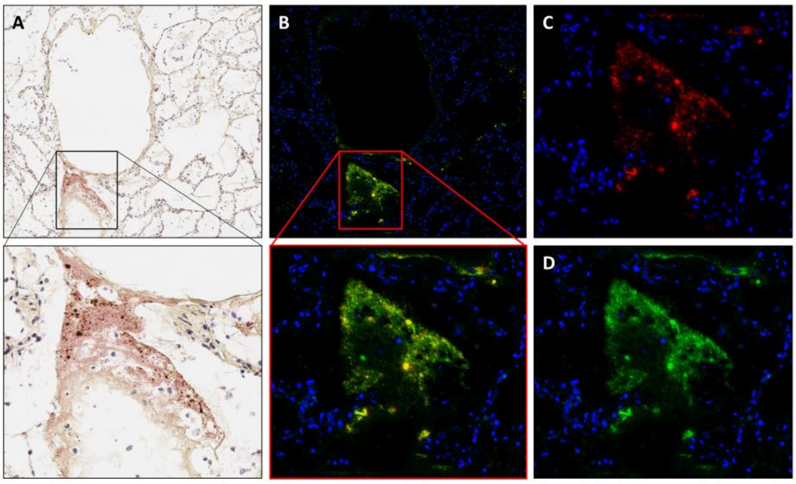

Supplementary Figure S1. (A) IHC and RNAScope Dual-plex DAB x10, with exploded view x20. (B) IHC and RNAScope dual-plex mIF x10, with exploded view x20. (C) mIF single channel displaying RNAScope only (Red) with DAPI counter stain (Blue) x20. (D) mIF single channel displaying IHC only (Green) with DAPI counter stain (Blue) x20.

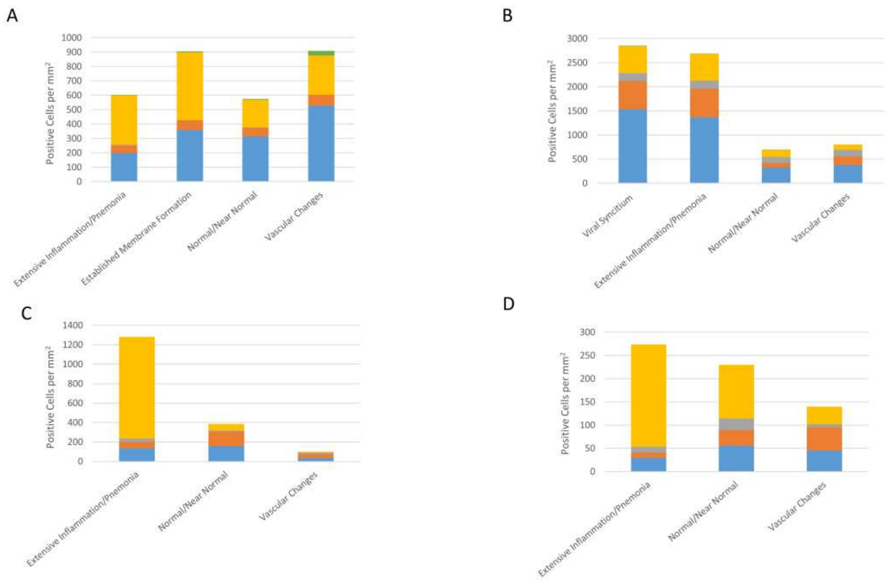

Supplementary Figure S2. (A) Case 1. (B) Case 2. (C) Case 3. (D) Case 4. CD3 – blue, CD4 – orange, CD8 – grey, CD68 – yellow, CD20 – green.

Multiplex Panel	Biomarker	Clone	Source	Catalogue Number	Epitope Retrieval	Dilution	Incubation	Detection Chemistry
**Panel 1**	CD3	2GV6	Ventana	5278422001	ER1 30 minutes	1 in 6	30 minutes at ambient	Opal 520
**Panel 1**	CD4	SP35	Ventana	5552737001	ER1 30 minutes	1 in 4	30 minutes at ambient	Opal 620
**Panel 1**	CD20	L26	Dako	M0755	ER1 30 minutes	1 in 400	30 minutes at ambient	Opal 570
**Panel 2**	PD-L1	SP263	Ventana	740-4907	ER2 20 minutes	1:2	30 minutes, ambient	Opal 520
**Panel 2**	CD8	C8/144B	Dako	M7103	ER2 20 minutes	1 in 400	30 minutes, ambient	Opal 690
**Panel 2**	CD68	514H12	Novocastra	NCL-L-CD68	ER2 20 minutes	1 in 200	30 minutes at ambient	Opal 570
**Panel 2**	CK	AE1/AE3	Dako	M3515	ER2 20 minutes	1 in 100	30 minutes at ambient	Opal 620

Table S1 – Antibody details and optimised retrieval methods
